# P53 Mutation Induces Epithelial-to-Mesenchymal Transition (EMT) Associated with Stem Cell Properties and Tumorigenesis in Fallopian Tube Cells

**DOI:** 10.3390/cancers17203317

**Published:** 2025-10-14

**Authors:** Kholoud Alwosaibai, Barbara C. Vanderhyden, Fatimah A. Alsaffar, Salma Alamri, Abdulaziz A. Almotlak

**Affiliations:** 1 Biomedical Research Department, Research Center, King Fahad Specialist Hospital-Dammam, Eastern Health Cluster, Dammam 32253, Saudi Arabia; 2 Departments of Cellular and Molecular Medicine, University of Ottawa, Ottawa, ON K1H 8M5, Canada; 3 Cancer Therapeutics Program, Ottawa Hospital Research Institute, Ottawa, ON K1H 8L6, Canada; 4 Department of Obstetrics and Gynecology, University of Ottawa, Ottawa, ON K1H 8L6, Canada; 5 Medical Laboratory Science Department, AlGhad College for Applied Medical Sciences, Dammam 32423, Saudi Arabia; 6 Department of Pharmacology, Imam Abdulrahman bin Faisal University, Dammam 31441, Saudi Arabia

**Keywords:** fallopian tube, oviduct, ovarian cancer, epithelial cells, stem cells, epithelial–mesenchymal transition, EMT, P53, BRCA1, CD44

## Abstract

**Background/Objectives**: Type II ovarian cancer, including high-grade serous carcinoma (HGSC), is genetically unstable and exhibits frequent mutations in the tumor suppressor genes. Mutations of *TP53* and *BRCA1* genes have been associated with HGSC, which has been suggested as a subtype that arises from the fallopian tube lesion called serous tubal intraepithelial carcinoma (STIC). Although *TP53* and *BRCA1* genes are well-known tumor suppressor genes, the actual effects of *TP53* and *BRCA1* mutations in enhancing the development of ovarian cancer initiated from STIC are poorly understood. **Methods:** In this study, we knocked out *Trp53* and *Brca-1* in epithelial cell clones derived from mice fallopian tube tissues (known as oviducts) and investigated the potential involvement of these two mutations in inducing cancer stem-like cells as cancer-initiating cells. **Results:** We have shown that the knockout of *Trp53* induced oviduct cells to undergo EMT and acquire stem cell characteristics. **Conclusions:** *Trp53* mutation may induce the early stage of precursor lesions formation at the distal end of the oviducts.

## Simple Summary

Ovarian cancer, particularly high-grade serous carcinoma, is one of the most aggressive and lethal gynecological cancers. Increasing evidence suggests that many of these tumors may begin in the fallopian tube, but the exact role of genetic mutations in this process is not fully understood. In this study, we explored how the loss of two well-known tumor suppressor genes, *Trp53* and *Brca1*, affect normal fallopian tube cells. We found that loss of *Trp53* caused these cells to grow faster, migrate more, and develop features of stem cells and epithelial-to-mesenchymal transition, changes that may allow them to act as early cancer-initiating cells. While BRCA1 loss alone did not strongly alter cell behavior, its combination with P53 loss further promoted cell growth and migration. These findings highlight the critical role of P53 in early ovarian cancer initiation and suggest that *Brca1* mutations may accelerate disease progression rather than initiate it.

## 1. Introduction

The origin of ovarian cancer is still a matter of debate. Researchers are investigating several types of cells and looking for factors from the reproductive tract that could contribute to the initiation of ovarian cancer, intending to identify mechanisms to inhibit this process for cancer prevention. Studies suggest that ovarian cancer originates from ovarian surface epithelial cells that migrate into the ovarian stroma during ovulation, forming inclusion cysts that may transform. This hypothesis was supported by evidence that various Hox genes could transform mouse ovarian surface epithelial cells (OSE) into cancers with different histology similar to those in human ovarian cancers [1,2]. More recently, it has been found that the markers of serous ovarian carcinoma closely match those expressed by the secretory cells of the fallopian tube, suggesting an alternative origin [3]. HGSC’s molecular analysis identified a gene expression common to the fallopian tube [4,5,6]. PAX2 and PAX8 are two proteins commonly expressed in the fallopian tube and HGSC but not in the OSE cells [6,7,8]. Moreover, lesions identified in the fallopian tube carry the same mutations of *TP53* as found in co-existing HGSC tumors [9]. Precursor lesions have also been detected in the distal end of the fallopian tubes in patients carrying *BRCA1* or *BRCA2* mutations [10,11]. These precursor lesions in the fallopian tube include stratified and atypical epithelial cells defined as serous tubal intraepithelial carcinoma (STIC) [12,13]. Thus, the current recommendation for women with *BRCA1* and *BRCA2* mutations is to consider bilateral salpingo-oophorectomy (removal of both ovaries and fallopian tubes) to reduce the risk of developing ovarian cancer [14,15,16].

It has been proposed that the close association between the ovary and fallopian tube in the female reproductive system may increase the possibility of cancerous fallopian tube cells exfoliating and implanting on the surface of the ovary. Furthermore, exfoliated epithelial cells from the fallopian tube to the surface of the ovary may also invaginate into the ovary to form inclusion cysts that could develop ovarian cancers. This model has received extensive support and led to investigations into mechanisms that might allow or promote the migration of epithelial cells to the surface of the ovary [17].

Several animal studies have provided evidence that fallopian tube lesions can initiate ovarian cancer, and the fallopian tube epithelium is therefore considered a potential cell of origin for serous carcinomas. One study demonstrated that double knockout of *Dicer* and *PTEN* in the oviducts of mice resulted in lesions at the distal end of the oviduct. These lesions spread and metastasized to the ovary and developed ovarian cancer. Surprisingly, removal of fallopian tubes prevented tumor formation, which supports the hypothesis of a fallopian tube origin of ovarian cancer [18]. A recent study in which a *Trp53* mutation was added to the *Dicer* and *PTEN* mutations revealed that these mice developed HGSC in the fallopian tubes and the ovary. However, when the oviducts were removed, the deficient mice could still develop HGSC in the ovaries and metastasize to the peritoneal cavity [19], adding strength to the hypothesis that HGSC can arise from both the ovary and the fallopian tube [18,20]. However, someone could argue that since the oviducts were removed from the mice at two months of age, the oviductal epithelial cells (OVE) might have had an opportunity before then to migrate from the fimbria and implant on the surface of the ovary.

Recent findings from mouse models incorporating hereditary risk also implicate the fallopian tube as an origin for HGSC. It has been reported that oviductal secretory cells with *Brca1*, *Trp53*, and *PTEN* mutations developed fallopian tube lesions that metastasized to the ovaries and the peritoneal cavity to form HGSC in mice [21]. In humans, precursor lesions have been detected in the distal end of the fallopian tubes in patients carrying *BRCA1* or *BRCA2* mutations [10]. These precursor lesions in the fallopian tube include stratified and atypical epithelial cells, which have been defined as STIC [12].

Several recent studies support the view that the inclusion cysts are made of either OSE invaginations from the surface of the ovary or tubal epithelial cells invaginating from the ovarian surface after traveling from the fimbriae and implanting on the surface of the ovary [22,23]. It has been suggested that inclusion cysts formed by the tubal cells develop into HGSC, representing most serous cancers. In contrast, the inclusion cysts formed by OSE cells develop low-grade serous cancer (LGSC) [24].

Because of its recent implication in the origins of HGSC, the cellular origin of the inclusion cyst is now hotly debated. Thus, it has been hypothesized that junctional zone cells (transitional epithelial cells between the fallopian tube and the surface of the ovary) are prone to neoplastic transformation [25]. Consistent with that hypothesis, the junctional zone is enriched with label-retaining cells that express stem/progenitor cell markers such as CD44, ALDH, LGR5, and CD133 [26,27]. Further, the junctional zone cells have transformation potential when the tumor suppressor genes *TP53* and *Rb1* are inactivated, suggesting that the stem/progenitor cells in the junctional zone may be cells of origin for HGSC [26].

Whether junctional zone cells are the origin of ovarian cancer remains a controversial issue. However, some studies have suggested that they are fimbrial precursor cells since they acquire expression of the fallopian cell marker (PAX8) [8].

Since STICs are thought to arise from fallopian tube cell outgrowths that frequently have *TP53* mutation and these lesions have been detected in *BRCA-1* carriers [23,28,29], this study aimed to define the role of *TP53* and *BRCA-1* mutations in fallopian tube cells by characterizing the potential involvement in the regulation of stem-like cell proliferation and gene expressions that may be relevant to cancer-initiating cells. Herein provides evidence that the knockout of *TP53* and/or *BRCA-1* in OVE cells changes the cell behavior and cell expressions, including stem cell markers, which indicate involvement in ovarian cancer.

## 2. Methods

### 2.1. Experimental Animal

Twenty-five-day-old female FVB/N mice (The Jackson Laboratory, Bar Harbor, ME, USA) were housed with free access to food and water. All experimentations with mice were performed in accordance with the Canadian Council on Animal Care’s Guidelines for the Care and Use of Animals under a protocol approved by the University of Ottawa’s Animal Care Committee (no. ME-256). *Brca1^fl/fl^* [FVB;129-Brca1tm2Brn], *Trp53^fl/fl^* [FVB;129-Trp53tm1Brn], and *Trp53^fl/fl^/Brca1^fl/fl^* [FVB.129P2-Brca1tm1BrnTrp53tm1Brn] mice were obtained from the Mouse Models of Human Cancers Consortium Mouse Repository (National Cancer Institute, Rockville, MD, USA).

### 2.2. Cell Lines and Cell Culture

To generate primary cultures of oviductal epithelial (OVE) cells isolated from *Trp53^fl/fl^*, *Brca1^fl^*^/*fl*,^, and *Trp53^fl/fl^*/*Brca1^fl/fl^* mice, oviductal cells were isolated from 25-day-old female euthanized FVB/N mice *Trp53^fl/fl^* (designated P53-flox), *Brca1^fl/fl^* (designated BRCA1-flox), and *Trp53^fl/fl^*/*Brca1^fl/fl^* (designated P53/BRCA1-flox). Oviducts were rinsed with phosphate-buffered saline (PBS), minced, and incubated with 0.25% trypsin (Invitrogen, Thermo Fisher Scientific, Waltham, MA, USA) for 30 min at 37 °C in 5% CO_2_ to dissociate epithelial cells. Fragments containing cells were cultured in OVE medium consisting of minimal essential medium (MEM; (GE Healthcare, Chicago, IL, USA) supplemented with 4% fetal bovine serum (PAA Laboratories GmbH, Pasching, Austria), 0.01 nM estradiol (Sigma-Aldrich, St. Louis, MO, USA), 5 U/mL penicillin–streptomycin (Sigma-Aldrich, St. Louis, MO, USA), 0.1 µg/mL gentamicin (Invitrogen, Thermo Fisher Scientific, Waltham, MA, USA), 0.02 µg/mL epidermal growth factor (R&D Systems, Inc., Minneapolis, MN, USA), and 1 µg/mL insulin–transferrin–selenite supplement (Roche Diagnostics GmbH, Mannheim, Germany). Cultures were maintained for up to two weeks until cells adhered and spread. Adherent cells were then collected by trypsinization, replated as single cells, and expanded into independent colonies. Each clone was numbered and passaged as a new OVE-derived cell line. The epithelial phenotype was confirmed by E-cadherin and CK19 expressions. Four main clones were derived to perform this study: BRCA1-Clone 1-flox, BRCA1-Clone 2-flox, P53-flox, and P53/BRCA1-flox.

### 2.3. Adenovirus Infection to Inactivate Tumor Suppressor Genes

OVE cells that were isolated from *Trp53^fl/fl^*, *Brca1^fl/fl^*, and *Trp53^fl/fl^/Brca1^fl/fl^* were infected in a serum-free medium with 2000 pfu/cell of Adenovirus expressing Cre recombinase (AdCre) or adenovirus expressing GFP (GFP). The infected cells were incubated at 37 °C in a serum-free medium, and after one hour, serum was added to 10% to terminate the infection. Six hours later, the adenovirus medium was removed, the cells were washed three times with PBS, and fresh OVE medium was added. The infected cells were washed with PBS every 12 h for three days, and fresh media was added at each wash.

### 2.4. Detection of Recombination Using PCR

OVE cells infected with AdCre and GFP were incubated until they recovered after three cell passages and then dissociated with trypsin. Genomic DNA was extracted using the Extract-N-Amp Tissue PCR kit (Sigma-Aldrich, St. Louis, MO, USA) following the manufacturer’s protocol. Recombination of the *Trp53* floxed region was detected by amplification of a 612 bp band using primers for *Trp53* intron1 forward (5′ CAC-AAA-AAA-CAG-GTT-AAA-CCC-AG 3′) and *Trp53* intron10 reverse (5′ GAA GAC AGA AAA GGG GAG GG 3′). Unrecombined alleles were detected by amplification of a 370 bp band using the primers for *Trp53* intron1 forward (5′ CAC-AAA-AAA-CAG-GTT-AAA-CCC-AG 3′) and *Trp53* intron1 reverse (5′ AGC ACA TAG GAG GCA GAG AC 3′). The deletion of floxed exons 5–13 of *Brca1* was detected by amplification of a 600 bp band using primers for *Brca1* intron 4 forward (5′ TAT CAC CAC TGA ATC TCT ACC G 3′) and *Brca1* intron 13 reverse (5′ TCC ATA GCA TCT CCT TCT AAA C 3′). The unrecombined allele was detected by amplification of a 592 bp band using primers for *Brca1* intron 4 forward (5′ TAT CAC CAC TGA ATC TCT ACC G 3′) and *Brca1* intron4 reverse (5′ GAC CTC AAA CTC TGA GAT CCA C 3′). The PCR amplification was performed in 10 μL volumes of 1 μg of genomic DNA, 1X REDExtract-N-AmpTM (Sigma-Aldrich, St. Louis, MO, USA), and one nmol of each primer. PCR conditions were initially denatured at 94 °C for 3 min, then 30 cycles of 94 °C for 30 s, 56 °C for 30 s, and 72 °C for 60 s, followed by 72 °C for 10 min. The PCR products were separated on a 1% agarose gel with Tris-acetate-EDTA buffer.

### 2.5. Proliferation Assay

The proliferation assay was performed to determine the effect of inactivating *Trp53* and/or *Brca1*. The recombined and unrecombined cells were plated at a density of 5 × 10^3^ cells/mL in 24-well plates. Triplicate samples of each group of cells were trypsinized and counted daily using a Vi-CELL XR Cell Viability Analyzer (Beckman Coulter, Inc., Brea, CA, USA).

### 2.6. Migration Assay

Cell migration was evaluated using a scratch-wound assay. Briefly, confluent cell monolayers were scratched with a pipette tip, washed with PBS to remove detached cells, and replenished with fresh OVE medium. The scratches were labeled and imaged at 0 h, then incubated at 37 °C. Wound closure was monitored by imaging at 6 and 18 h. Gap closure was quantified using ImageJ software v1.53t (NIH, Bethesda, MD, USA).

### 2.7. Sphere Formation Assay

The sphere formation assay was performed as reported previously [27]. The sphere formation capacity was assessed by the sphere size and sphere numbers. Spheres with diameters of more than 60 µm were counted and measured. The percentage of spheres exceeding this diameter was determined for each group of OVE cells and analyzed using ImageJ software v1.53t (NIH, Bethesda, MD, USA).

### 2.8. Colony Formation Assay in Soft Agar

Recombined and unrecombined OVE cells, breast cancer cell line (MCF7), and Spontaneously Transformed Ovarian Surface Epithelial (STOSE) (positive control) were dissociated from adherent culture, and single cells were collected after passing the cells through a 40-µm cell strainer (Becton Dickinson, Franklin Lakes, NJ, USA). The 24-well culture plates for the colony formation were prepared with 500 µL of a base layer of agar consisting of 2% ultrapure low-melting-point agarose (Life Technologies, Carlsbad, CA, USA) and 2X D-MEM medium in a ratio of 1:1. The agar layer was solidified at 4 °C for 15 min and warmed at 37 °C for 15 min before adding the top layer of 500 µL of an equal volume-mixture of 1% agarose and 2× D-MEM medium containing the single cell suspension (1 × 10^5^ cell/mL). The cells were then incubated at 37 °C for 4–6 weeks, and colonies were visualized and counted using the EVOS XL imaging system (Life Technologies, Carlsbad, CA, USA).

### 2.9. Gene Expression Analysis

Quantitative real-time PCR (qPCR) was performed to determine the gene expression for all OVE clones, as described previously [27], using primers and probes listed in the Supplementary Table S1. The gene expression levels for OVE and stem cell markers are relatively analyzed in relation to the endogenous control Ppia or TBP.

### 2.10. Western Blot Analysis

Cell lysates were prepared and subjected to Western blotting as previously reported [27]. Proteins transferred to nitrocellulose membranes were incubated with primary antibodies against PAX2 (mouse, 1:20,000; Santa Cruz Biotechnology, Dallas, TX, USA), P53 (mouse, 1:10,000; Invitrogen, Thermo Fisher Scientific, Waltham, MA, USA), or BRCA1 (rabbit, 1:200; Santa Cruz Biotechnology, Dallas, TX, USA). Secondary detection employed horseradish peroxidase-conjugated antibodies, either anti-rabbit (1:10,000; Abcam, Cambridge, UK) or anti-mouse (1:10,000; Sigma-Aldrich, St. Louis, MO, USA). Signals were visualized using enhanced chemiluminescence (Clarity Western ECL Substrate; (Bio-Rad, Hercules, CA, USA) and recorded with a FluorChem FC2 imaging system (Alpha Innotech, San Leandro, CA, USA).

### 2.11. Flow Cytometry for SCA-1 Expression

The dissociation of OVE cells was performed using non-enzymatic cell dissociation solution (MULTICELL, Seoul, Republic of Korea) and then the cells were filtered through a 40-µm strainer. Single-cell suspensions were incubated with mouse SCA-1-FITC antibody (1:11; Milteny Biotec, Bergisch Gladbach, Germany) for 15 min at 4 °C. Excess antibody was removed by washing, and SCA-1–positive cells were quantified using a MoFlo cell sorter (Dako Cytomation, Fort Collins, CO, USA).

### 2.12. Immunohistochemistry

Fixed Ovarian cancer tissues, including STIC lesions, were collected from the pathology department after the approval by the Institutional Review Board (IRB) (IRB# ONC0340). The paraffin-embedded blocks containing the cancer tissues were cut on serial sections with a thickness of 4 μm. Serial sections for each block were stained with antibodies against CD44 (Roche Diagnostics GmbH, Mannheim, Germany), P53 and Ki67 (Leica Biosystems, Wetzlar, Germany). To determine CD44 expression, automated staining was performed using the Ventana Benchmark instrument (Roche Diagnostics GmbH, Mannheim, Germany), following the manufacturer’s instructions. For P53 and Ki67, the manual immunohistochemistry protocol was used. The tissue was deparaffinized and rehydrated in graded ethanol. For antigen retrieval, the tissues were heated at 90 °C using Novocastra Epitope Retrieval Solution, pH 9 (Leica Biosystems, Wetzlar, Germany). Tissue sections were blocked with protein block serum-free solution (Leica Biosystems, Wetzlar, Germany) for 1 h and then incubated for 1 h with mouse anti-P53 and mouse anti-Ki67. After washing in PBS, the tissues were incubated with anti-mouse or anti-rabbit secondary antibodies. The protein detection was performed using a Novolink polymer detection system (Leica Biosystems, Wetzlar, Germany) following the manufacturer’s instructions, and the immunoreactivity was imaged using the PreciPoint M8 system and viewpoint light software v1.4. (PreciPoint GmbH, Freising, Germany).

Immunohistochemical protein expressions were scored by defining the percentage of positive cells out of the whole tissue. The CD44 immunostaining was evaluated as positive when cytoplasmic membrane staining was observed and tumor cells expressed more than 50% out of all tumor sections. The P53 immunostaining was assessed as negative (less than 10% are reactive cells) and positive (equal or more than 10% are reactive cells). However, lesions with TP53 mutation were classified as P53 signatures, serous tubal intraepithelial lesions (STILs), or serous tubal intraepithelial carcinomas (STICs) based on P53 and Ki-67 immunostaining patterns combined with cellular morphology. A P53 signature was recognized as more than 12 consecutive epithelial cells with strong nuclear P53 staining, low proliferative activity (Ki-67 <10%), and no morphological atypia. STILs were defined as P53 accumulation in more than 20 epithelial cells, some nuclear atypia, and an intermediate Ki-67 index (10–40%). STICs were defined as extensive epithelial involvement, marked nuclear and architectural abnormalities, diffuse strong P53 positivity, and high proliferative activity [11]. To quantify the percentage of positive staining area, representative IHC images from each patient were analyzed using ImageJ (NIH, Bethesda, MD, USA).

### 2.13. Statistical Analyses

Statistical analyses were performed using GraphPad Prism v10.0 (GraphPad Software, San Diego, CA, USA). A Student’s *t*-test (two groups) or ANOVA with Tukey’s post-test (multiple groups) was used to determine statistical significance (*p* < 0.05). Error bars represent the standard error of the mean.

## 3. Results

### 3.1. Isolation and Characterization of OVE Cells from Mice with Conditional Expression of Trp53 and/or Brca1

**Figure 1:**
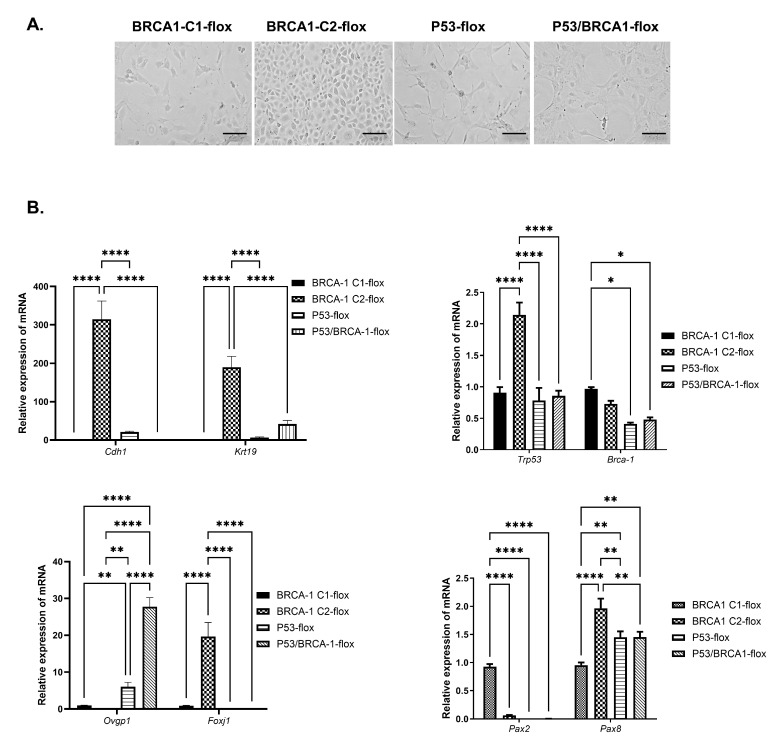
OVE clonal cells have different cell morphology and gene expressions. (**A**) Microphotographs of OVE clonal cells show the epithelial morphology of BRCA1-C2-flox cells and the more mesenchymal morphology of the other three cell lines. Scale bar: 100 μm. (**B**) qPCR analysis shows variable gene expression for epithelial markers (*Cdh1* and *Krt19*), tumor suppressor genes (*Trp53* and *Brca1*), and OVE markers (*Ovgp1, Foxj1, Pax2, and Pax8*) among the four cell lines. In line with their morphology, BRCA1-C2-flox clonal cells express higher levels of transcripts for epithelial markers (*Cdh1* and *Krt19*) and markers of ciliated cells (*Foxj1*) relative to most of the other cell lines. P53-flox and P53/BRCA1-flox cells express lower levels of mRNA for epithelial markers (*Cdh1* and *Krt19*) and elevated levels of secretory cell markers (*Ovgp1*). *Pax8* mRNA is equally expressed in all OVE clonal cells, whereas *Pax2* is highly expressed in the BRCA1-C1-flox cells. Analyses were performed using multiple *t*-test analyses for three independent experiments. * *p* < 0.01, ** *p* < 0.001, and **** *p* < 0.00001.

To determine the effect of *Trp53* and *Brca1* mutations on OVE cell behavior and gene expression, clones of OVE cells were established from transgenic mice having flanked loxP sites located in the *Trp53* or/and *Brca1* genes. The loxP sites in the *Trp53* gene are located between exons 1 and 10, and the loxP sites in the *Brca1* gene are located between exons 4 and 13. In both cases, Cre-mediated deletion should result in the excision of the DNA segments flanked by the loxP sites. After a few passages, the morphology of the clonal cells was assessed, and each clone was found to have different morphologies (Figure 1A).

**Figure 2:**
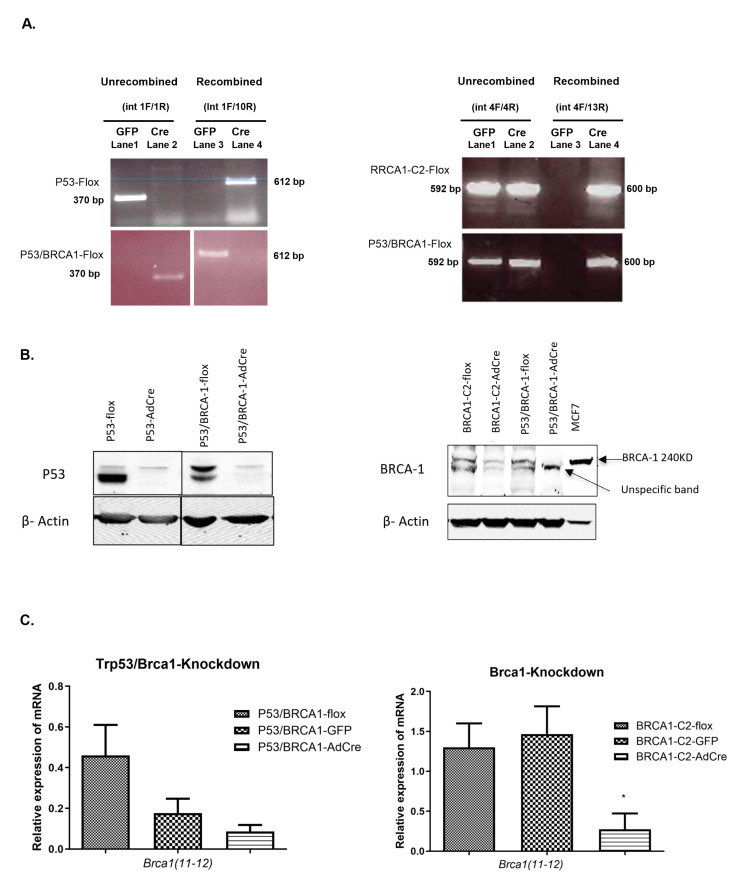
Conditional inactivation of *Trp53* and *Brca1* in OVE cells. (**A**) Gel electrophoresis shows the recombination of both *Trp53* and *Brca1* DNA after exposure to AdCre. Recombination of the *Trp53* floxed region was detected in 612 bp band and unrecombined alleles were detected in 370 bp band (**left panel**). The deletion of *Brca1* floxed region was detected in 600 bp band and the unrecombined allele was detected in 592 bp band (**right panel**). (**B**) Protein analysis shows the decreased expression of BRCA1 protein in BRCA1-C2-AdCre and P53/BRCA1-AdCre. (**C**) qPCR analysis for *Brca1* mRNA shows decreased levels of *Brca1* gene expressions in P53/BRCA1-AdCre and BRCA1-C2-AdCre, MCF7 was used as the positive control for BRC-1 protein and gene expressions. Analyses were performed using one-way ANOVA for three independent experiments. The blue line appearing in Figure 2A is an artifact generated by the gel imaging system. It does not represent a label or a feature of the sample and should be disregarded. * *p* < 0.01.

To characterize the new OVE clonal cells, the expression of several genes encoding epithelial and OVE markers, as well as the tumor suppressor genes *Trp53* and *Brca1* were determined (Figure 1B). P53-flox and P53/BRCA1-flox clonal cells expressed mRNA encoding the epithelial markers *Krt19* and *Cdh1*. However, the BRCA-Clone2-flox (BRCA-C2-flox) expressed the highest levels of *Krt19* and *Cdh1* mRNA since this clone tends to have more epithelial cell morphology than other clones. The secretory cell marker (*Ovgp1*) was highly expressed, whereas expression of the ciliated cell marker *Foxj1* was lower, potentially contributing to a more mesenchymal morphology. In addition, we investigated the expression of the transcription factors Pax8 and Pax2, which are commonly found in OVE cells in vivo. *Pax8* mRNA was evenly expressed in both OVE clonal cells, but the expression of *Pax2* mRNA in P53-flox clonal cells was barely detectable, whereas P53/BRCA1-flox cells expressed no detectable *Pax2* mRNA. Assessment of the expression of *Trp53* and *Brca1* mRNA confirmed detectable levels in all OVE clonal cells. To determine the effect of the loss of *Trp53* and *Brca1* on OVE cells, we treated OVE clonal cells with AdCre to inactivate *Trp53* and/or *Brca1*. The control cells were treated with GFP. PCR was used to confirm DNA recombination at the loxP sites, and it was shown that in *Trp53*, exons 1 to 10 were deleted efficiently. However, recombination of the *Brca1* gene at exons 4 to13 was less efficient, retaining detectable unrecombined populations of BRCA-Clone1-flox (BRCA1-C1-flox), BRCA1-C2-flox, and P53/BRCA1-flox cells, even after treatment with AdCre (Figure 2A).

For further confirmation, Western blots and quantitative qPCR were performed to determine the levels of BRCA1 protein and encoded mRNA. Western blots showed decreased BRCA1 expression in BRCA1-C2 and P53/BRCA1 cells after treatment with AdCre (Figure 2B). For qPCR, primers for exons 11–12 of the *Brca1* gene were used, which should be deleted after AdCre treatment. *Brca1* mRNA encoded by these exons was significantly down-regulated after AdCre treatment in BRCA1-C2 clonal cells and modestly decreased in P53/BRCA1 clonal cells (Figure 2C). However, in BRCA1-C1-AdCre cells, recombination was detected in genomic DNA, but *Brca1* transcripts did significantly decrease in a subpopulation of P53/BRCA1 clonal cells under study (The uncropped blots and molecular weight markers are shown in Supplementary Figure S3). 

### 3.2. Loss of Trp53 Alone or Combined with Brca1 Induced OVE Cells to Undergo an Epithelial–Mesenchymal Transition

Cell morphology was investigated after AdCre treatment to assess the consequences of *Trp53* and *Brca1* inactivation in OVE cells and compared with those treated with GFP. *Brca1* inactivation significantly changed cell morphology to more mesenchymal than control cells (Figure S2). In contrast, OVE cells with combined mutation of *Trp53* and *Brca1* or with *Trp53* mutation alone did not show any changes in cell morphology, perhaps because these cells already had a more mesenchymal phenotype.

**Figure 3:**
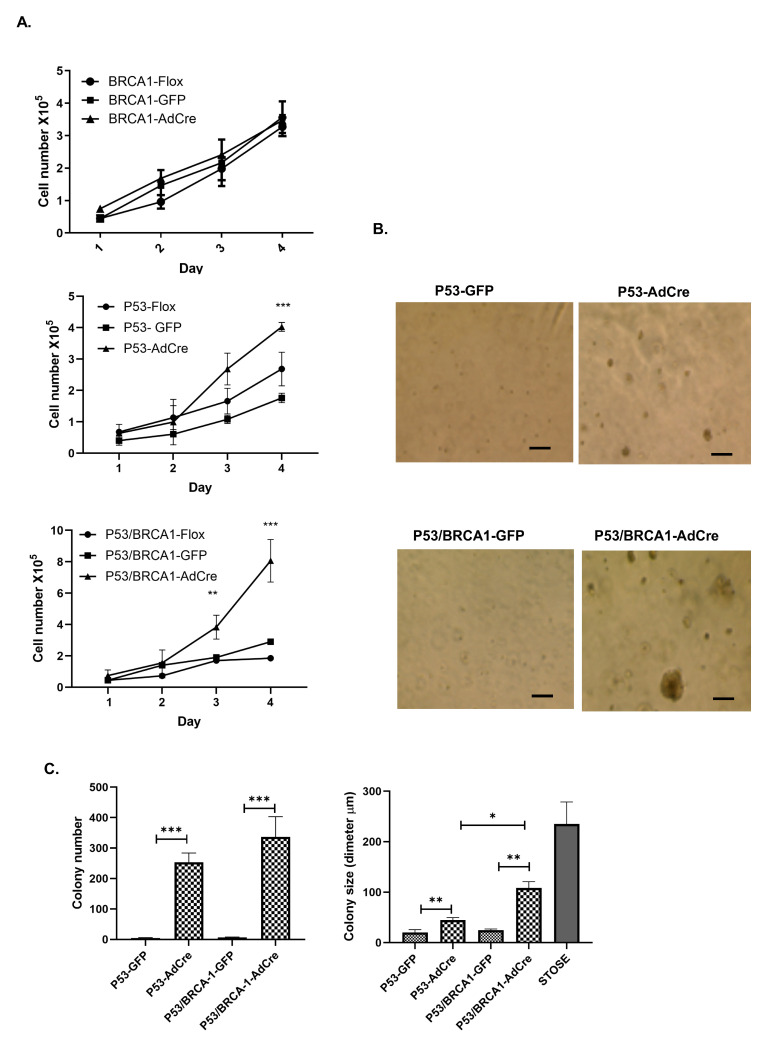
Loss of P53 alone or in combination with BRCA1 increases cell proliferation and colony formation. (**A**) Proliferation assays show significant increases in cell proliferation after *Trp53* knockout with and without *Brca1* inactivation, but not with *Brca1* inactivation alone. Analyses were performed using one-way ANOVA for three independent experiments. (**B**) Microphotographs show the capacity for colony formation after *Trp53* knockout and after *Trp53* and *Brca1* knockout; the scale bar is 100 μm. (**C**) Quantitative analysis of the number and size of formed colonies, STOSE is a positive control for colony formation assay. Analysis was performed using multiple *t*-test analysis for three independent experiments, * *p* < 0.01, ** *p* < 0.001 and *** *p* < 0.0001.

To determine if inactivation of *Brca1* and *Trp53* is associated with any other changes in cell behavior, cell proliferation for the three cell lines with *Brca1* or *Trp53* mutation alone or in combination was analyzed. The proliferation of OVE cells with reduced BRCA1 did not change compared to their control. However, *Trp53* inactivation in OVE cells, with or without loss of *Brca1*, significantly increased cell proliferation within four days (Figure 3A). Likewise, colonies were formed in soft agar when *Trp53* was inactivated in OVE cells, whereas the single inactivation of *Brca1* did not alter the cell capability to form colonies in soft agar. Mutations in both *Trp53* and *Brca1* enabled the cells to form large colonies (Figure 3B,C). Thus, these data indicate that loss of *Trp53* alone or combined with loss of *Brca1* increases cell proliferation and colony formation, whereas loss of *Brca1* alone only changes cell morphology.

**Figure 4:**
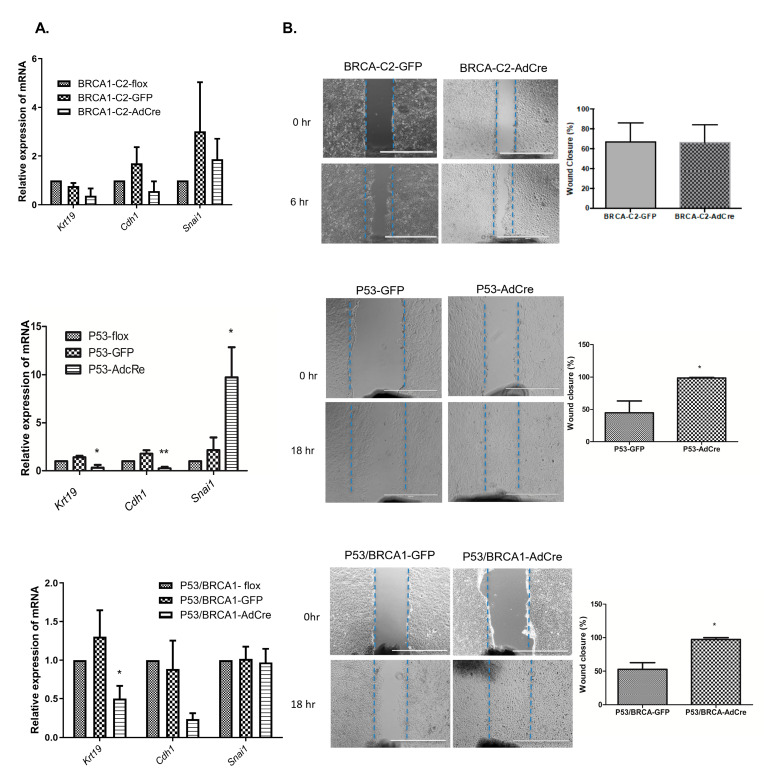
Loss of *Trp53* alone or in combination with *Brca1* induced OVE cells to undergo epithelial–mesenchymal transition. (**A**) qPCR analysis shows modest, but not significant, down-regulation of epithelial marker transcripts after the inactivation of *Brca1*. In contrast, the inactivation of *Trp53* decreased the mRNA for epithelial markers and increased the expression of the EMT marker, Snail. The combined inactivation of *Trp53* and *Brca1* decreased the abundance of transcripts for epithelial markers but did not affect levels of Snail mRNA. Multiple one-way ANOVA statistical tests were performed for three independent experiments, * *p* < 0.05 and ** *p* < 0.01. (**B**) Photomicrographs and wound-healing analysis showed enhanced cell migration with loss of *Trp53*, with and without concurrent loss of *Brca1*. The blue lines indicate the wounds size at the start point. scale bar is 1000 μm. Results represent three independent replicates, and statistical analysis was performed using multiple *t*-tests.

BRCA1-C2-flox cells have an epithelial morphology and high expression of the epithelial markers E-cadherin (*Cdh1*) and CK19 (*Krt19*) (Figure 1B). Inactivation of *Brca1* with AdCre treatment did not change the abundance of transcripts encoded by *Krt19* and *Cdh1*. Although the loss of *Brca1* changed the cell morphology to become more mesenchymal, the mRNA expression for the EMT marker, Snail, was not altered by the loss of *Brca1*, suggesting that the reduction in BRCA1 was not sufficient to induce EMT in these OVE cells. In contrast, *Trp53* knockout significantly down-regulated the mRNA encoding the epithelial markers CK19 and E-cadherin and up-regulated Snail transcripts, indicating that loss of *Trp53* effectively induces EMT in OVE cells. Interestingly, the combined mutation of *Trp53* and *Brca1* decreased the epithelial markers but did not increase Snail transcripts (Figure 4A).

To determine if OVE cells gained another indicator of EMT, their migration ability was analyzed after the inactivation of *Trp53* and/or *Brca1*. The migration of OVE cells was not affected by the loss of BRCA1 alone; however, the inactivation of *Trp53* alone or in combination with *Brca1*, increased the migration of OVE cells toward the wound gap. This enhanced migration was evident within 18 h of creating the scratch wound. It was, therefore, not likely a result of increased cell proliferation, as the slow doubling time of these cells does not reveal any differences in growth rates for at least 48 h (Figure 4B).

### 3.3. Loss of Trp53 Enhances Sphere-Forming Capacity and Expression of the Stem Cell Markers CD44 and SCA-1

**Figure 5:**
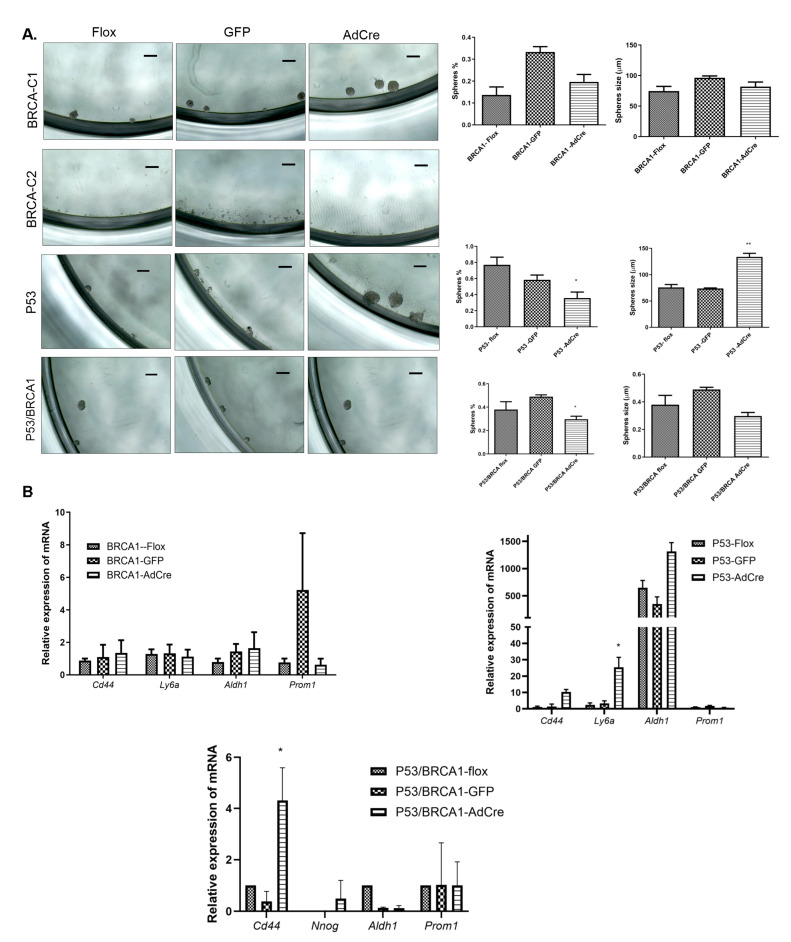
Analysis of stem cell markers following inactivation of *Brca1*, *Trp53*, and *Trp53*/*Brca1*. (**A**) Sphere formation assays in suspension culture for two weeks show that BRCA1-C1 clonal cells could form spheres, but BRCA1-C2 clonal cells could not form spheres with or without *Brca1* knockout. Corresponding bar graphs (**right panel**) show the percentage and size of formed spheres for each clone and condition, indicating no significant effect of *Brca1* loss on sphere formation capacity. In contrast, the loss of *Trp53* alone increased the size of the spheres, and combined loss of *Trp53* and *Brca1* increased the number of spheres indicating an increase in sphere formation capacity. The scale bar is 100 µm. Analysis was performed using one-way ANOVA for the sphere percentage and for the average sphere size obtained from three independent experiments. (**B**) qPCR analysis shows that loss of *Trp53* significantly increased the stem cell markers Sca-1 and CD44 in P53-AdCre and P53/BRCA1-AdCre clones, respectively. Multiple one-way ANOVA statistical tests were performed for three independent experiments, * *p* < 0.05 and ** *p* < 0.001.

To investigate if the inactivation of *Trp53* or/and *Brca1* and the associated shift to a more mesenchymal phenotype would also alter the stem/progenitor-like state of the OVE cells, sphere formation assays were performed, and the sphere sizes were determined. Inactivation of *Brca1* did not induce sphere-forming capacity in these cells. OVE clones (BRCA1-C2) that are epithelial and express high levels of E-cadherin, CK19, and markers of ciliated cells (FoxJ1), were less able to form spheres, whereas OVE clones with less epithelial morphology, lower levels of E-cadherin and CK19, and higher levels of the secretory marker OVGP, were more capable of forming spheres in suspension (Figure 5A).

Assessment of stem cell markers gene expressions indicated that BRCA1-C1-AdCre cells did not exhibit elevated levels of stem cell markers compared to parental cells, whereas BRCA1-C2 cells displayed notably low expression of these stem cell genes *CD44*, *Ly6a* (gene encoded for SCA-1), and *Aldh1*, and inactivation of *Brca1* did not affect their expression (Figure 5B). The P53-flox OVE cells formed spheres in suspension, and *Trp53* knockout did not change the number of spheres; however, the loss of *Trp53* alone or combined with *Brca1* mutation increased sphere formation capacity, indicating the enhanced ability of OVE stem cells to proliferate in suspension compared to the control cells (Figure 5A). To determine if loss of *Trp53* increased the expression of stem cell genes, qPCR was performed. Sca-1 (*Ly6a*) and CD44 mRNA levels were significantly up-regulated after loss of *Trp53* (Figure 5B).

### 3.4. Trp53 Mutation Enhances the Stem Cell-Positive Population Associated with Tumorigenesis

**Figure 6:**
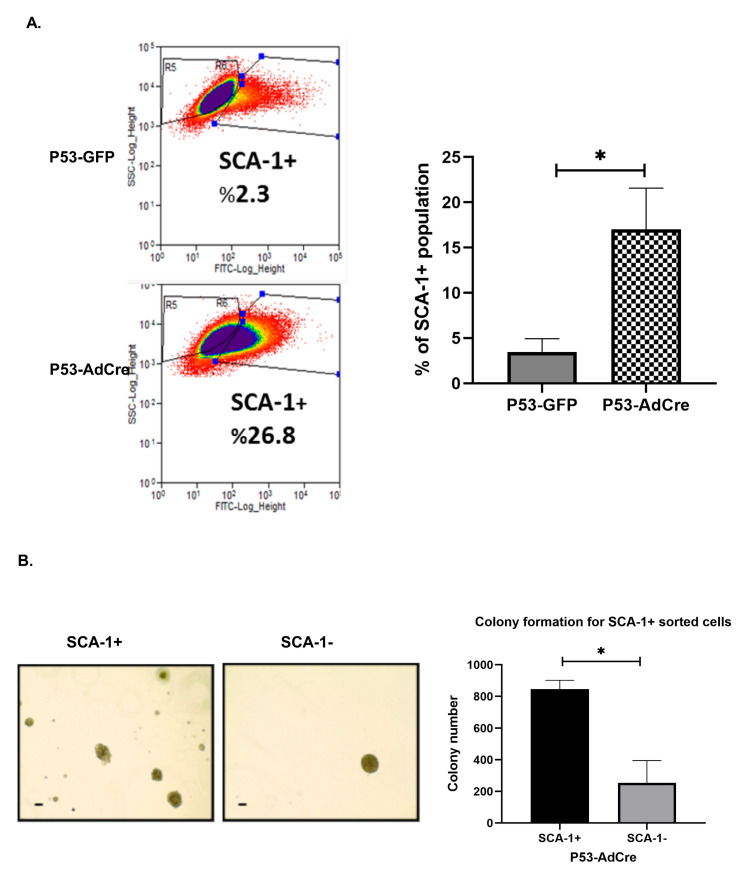
Inactivation of *Trp53* increases the SCA-1+ population. (**A**) Quantitative analysis of the SCA-1 positive fraction with *Trp53* mutation analyzed by flow cytometry. (**B**) Colony formation in soft agar for SCA-1 positive cells sorted by FACS from OVE with and without *Trp53* mutation (left panel), and quantitative analysis for the formed colonies by SCA-1 positive and SCA-1 negative populations for three independent experiments (right panel). Scale bar is 100 µm, * *p* < 0.01.

Since *Trp53* mutation was associated with induced colony formation and increased Sca-1 mRNA expression, we investigated if SCA-1 positive populations are associated with the ability to form colonies. We analyzed the proportion of SCA-1 positive OVE cells with and without *Trp53* knockout and found that the fraction of SCA-1 positive cells was highly up-regulated with *Trp53* mutation (Figure 6A). To determine if SCA-1 is associated with the ability to form colonies, positive and negative populations from OVE cells with and without *Trp53* mutation were plated in soft agar. SCA-1 positive cells sorted from OVE cells with *Trp53* knockout formed more colonies than the SCA-1 negative population (Figure 6B).

**Figure 7:**
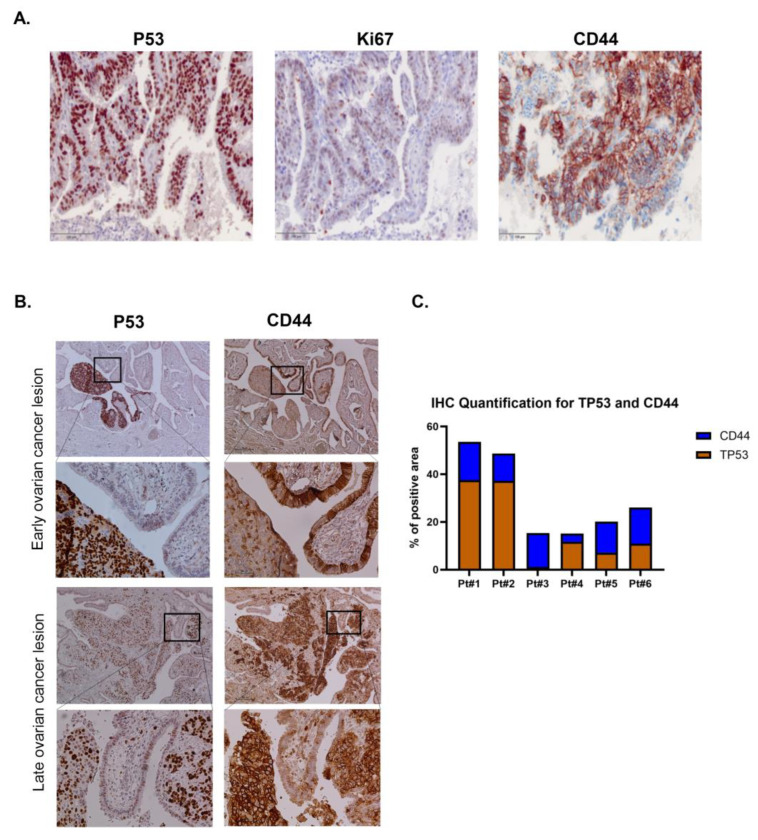
*TP53* mutation in serous tubal intraepithelial carcinoma increased CD44 expression in human tissues. (**A**) Immunohistochemistry staining for serial sections from human ovarian cancer tissues show STIC with *TP53* mutation and highly proliferative cancer cells expressing Ki67, co-localized with stem cell populations expressing CD44. (**B**) P53 signature in STIC is associated with low CD44 expression in early fallopian tube lesion and high expression in late fallopian tube lesion. (**C**) Quantification of immunohistochemical staining for CD44 and P53. Bar graph shows the percentage of CD44 and P53 positive cells in patient samples, based on quantitative analysis of IHC staining. Scale bar is 100 µm.

Building on previous findings, we extended our analysis to assess CD44 expression in STIC lesions characterized by P53 signatures. We found that the early lesion of STIC with P53 signature has less expression of CD44 in the lesion but has high expression of CD44 in the adjacent tissue. In contrast, the late lesion of STIC that has P53 signature is associated with CD44 expression. (Figure 7). Precisely, the expression of P53 and the stem cell marker is mutually exclusive at the early fallopian tube lesions and co-expressed in late tumor lesions (Supplementary Figure S1).

## 4. Discussion

Gene mutations of *Trp53* and *Brca1* in OVE cells were investigated to study the molecular mechanisms that trigger STIC formation. We found that loss of P53, with and without loss of BRCA1, increased cell proliferation and colony formation. In addition, the loss of P53 induced a shift to a more mesenchymal morphology in OVE cells and increased cell migration. Further, loss of P53 increased OVE stemness by increasing the sphere formation capacity and stem cell markers. Loss of P53 up-regulated the expression levels of the stem cell markers SCA-1 in mouse and CD44 in the human, which indicates that *Trp53* mutation in fallopian tube cells can promote the creation or amplification of EMT-associated stem cells.

These results agree with previous studies that reported that *Trp53* mutation is an inducer of EMT and stemness. That study showed the down-regulation of E-cadherin and up-regulation of the mammary stem cell population (CD24−/CD44+) after the loss of P53 [30], while in hematopoietic cells, loss of P53 increased SCA-1 and c-kit [31]. In addition, loss of P53 in the mammary gland increased stem cell self-renewal by increasing mammosphere number and size, suggesting that loss of P53 may increase the symmetric divisions [32].

Since we found that loss of P53 increased the stem cell population that expressed SCA-1 and enhanced colony formation in vitro, and was associated with high expression of another stem cell marker (CD44) in vivo, stem cells expressing these two markers with loss of P53 may act as tumor-initiating cells, which could develop into the precursor lesions of STIC found in the fallopian tube fimbria. We have previously shown that spontaneously transformed mouse ovarian surface epithelial (MOSE) cells with aberrant expression of P53 have a Sca-1+ population that develops into palpable tumors faster than Sca-1− cells after intrabursal injection [33]. Additionally, the current study has demonstrated that human fallopian tube lesions with a P53 signature also exhibit CD44 expression. Interestingly, levels of CD44 were detected in both early and late lesions, with higher levels of CD44. The result of our study is in line with our previous findings of high CD44 expression in epithelial ovarian carcinoma tissues [34,35] and with Kar et al. [36], who found elevated levels of CD44 in malignant cases with P53 positive and recorded CD44 expression in epithelial ovarian carcinoma tissues. The expression of CD44 was correlated with the tumor grade, such as HGSC, endometrioid, mucinous, and Ki67 expression [37].

In ovarian cancer, loss of PAX2 expressions was found to be associated with P53 signature in the early lesion that started in the fallopian tube. The detection of STIC that carries P53 signature and loss of PAX2 has become an important marker for determining the origin of ovarian cancer [27,38,39,40,41]. To know if *TP53* mutation associated with loss of PAX2 can regulate stem cell population and tumor progression, we investigated the expression of P53 signature in ovarian cancer and lesions STIC. We found in our study that human ovarian cancer lesion in early stage (STIC) regulates stem cells population, which might enhance tumor progression. The detection of stem cells that express LGR5 and ALDH have been reported in ovarian cancer, and the expressions of the stem cell markers correlates positively with cancer stage [42]. As reported previously and as expected, we found that STIC lesions that lost PAX2 and acquired P53 signature expressed elevated levels of CD44 [27].

*BRCA1* mutation is a well-established risk factor for ovarian cancer and has been linked to a higher incidence of serous tubal intraepithelial carcinoma (STIC) [43]. To investigate whether, and how, BRCA1 loss contributes to STIC formation, we inactivated *Brca1* with and without concurrent *Trp53* loss. Although *Brca1* recombination in our study was not fully efficient and a complete knockout was not achieved, we observed a significant reduction in BRCA1 expression following AdCre treatment, supporting the validity of our findings. Our results showed that loss of BRCA1 alone did not affect cell proliferation or alter cellular phenotype. Consistent with this, BRCA1 loss in OVE cells did not influence any of the stemness-related characteristics we assessed. This outcome contrasts with findings in mammary epithelial cells, where *BRCA1* inactivation reduces differentiation and expands the stem/progenitor cell population [44,45].

Interestingly, the combination loss of P53 and BRCA1 in OVE cells enhanced cell proliferation, migration, and colony formation but did not significantly alter stemness characteristics. Accordingly, the *Brca1* mutation might restrict the EMT-stem cell induction triggered by the loss of P53. The reason for the lack of any notable neoplastic changes after *Brca1* inactivation is unclear, but several factors should be considered. Most importantly, AdCre treatment of the *Brca1^fl^/^fl^* and *Trp53^fl/fl^/Brca1^fl/fl^* OVE cells did not result in complete knockout of the *Brca1* gene. Multiple attempts to isolate clones with fully inactivated *Brca1* were unsuccessful, and it remains unclear whether only a fraction of the cells had DNA recombination or whether one allele is less amenable to Cre-mediated recombination. The latter seems unlikely since the proper loxP-flanked DNA sequence was confirmed by sequencing, and the *Brca1^fl/fl^* mice have been used successfully in previous studies [46,47]. Based on Knudson’s two-hit hypothesis [48], we speculate that loss of both alleles of the *Brca1* gene must occur to demonstrate the biological significance of its inactivation. It is also notable that the *Brca1^fl/fl^* clone 2 used in this study appeared to be derived from ciliated cells, since they have high expression of ciliated cell markers (FOXJ) [49]. It has been reported that secretory cells are the cells of origin of STIC and HGSC and initiation of ovarian cancer [3,21,50].

Tumorigenesis is a multi-step process that includes an accumulation of gene mutations, and *BRCA1* mutation alone is insufficient to initiate tumor development. *BRCA1* mutation has been linked with precursor lesion development in *BRCA1* carriers [51], but the developed lesions have been characterized by aberrant P53 expression or *TP53* mutations in several studies [52,53,54,55]. *Brca1* inactivation in mouse OSE cells in vivo was also insufficient to induce tumor formation but required additional inactivation of *Trp53* [47,56]. Notably, the inactivation of *Brca1* in OSE cells in vitro results in a modest decrease in proliferation and increased apoptosis [46]. Cultures of mouse embryonic fibroblasts (MEFs) with a deletion of exon 11 of *Brca1* displayed a senescence-like growth defect [57], which raises the additional possibility that OVE cells with mutant *Brca1* had growth defects or increased susceptibility to apoptosis that impaired long term survival.

Unfortunately, the relationship between *Brca1*, *Trp53* mutations, and PAX2 expression could not be investigated because some of the OVE clonal cells had low or no PAX2 expression. Additional clones must be generated for future studies to examine the interrelationships between *Trp53* and *Brca1* mutations and PAX2 expression. Interestingly, we have reported the ability of PAX2 to suppress the routine induction of P53 expression in MOSE cells after treatment with cisplatin and the expression of P53 in a mouse model of ovarian cancer, raising the possibility that in STIC, the loss of PAX2 may be a mechanism by which P53 expression is increased [40,58].

Our study has several limitations that should be acknowledged. First, there is intrinsic heterogeneity among the oviductal epithelial cell clones, which were derived through single-cell isolation. These clones exhibited variable morphologies and expressed distinct biomarkers indicative of either epithelial or mesenchymal-like phenotypes. Such heterogeneity reflects the natural cellular diversity of the fallopian tube epithelium, which comprises secretory, ciliated, and basal cells, but it also introduces complexity in interpreting the specific effects of *Brca1* or *Trp53* knockout. Second, the ciliated cell clone (BRCA1-C2) did not form spheres and lacked expression of stem cell markers, which limited our ability to perform sphere formation analysis in this background. For this reason, we focused on the mesenchymal-like BRCA1-C1 clone, which could form spheres; however, *Brca1* knockout in BRCA1-C1 did not significantly alter the sphere-forming capacity. Third, BRCA1-C1 did not consistently show a strong reduction in BRCA1 expression, which further constrained our ability to evaluate the full consequences of BRCA1 loss in this clone.

Together, these limitations underscore that both clonal phenotype and gene knockout efficiency can substantially influence experimental outcomes. They also emphasize the need for careful consideration of clonal variability before generalizing findings to the broader fallopian tube epithelium, where cellular heterogeneity and variable gene expression are expected.

## 5. Conclusions

Our study demonstrates that loss of P53 in oviductal epithelial (OVE) cells drives multiple tumorigenic features, including increased proliferation, migration, colony formation, epithelial-to-mesenchymal transition (EMT), and stemness, as evidenced by elevated SCA-1 and CD44 expression. These findings suggest that P53 deficiency may promote the emergence or expansion of EMT-associated stem cells, which could serve as tumor-initiating cells and contribute to the development of early STIC lesions in the fallopian tube. While BRCA1 loss alone did not significantly affect proliferation or stemness, its combination with P53 loss enhanced proliferation and migration, indicating a potential cooperative effect in tumor progression. Overall, our results highlight the central role of P53 in regulating EMT, stemness, and early ovarian tumor initiation, and suggest that *BRCA1* mutations may act primarily as facilitators rather than primary drivers of STIC formation.

## Data Availability

The datasets generated or analyzed in this study can be obtained from the corresponding author upon reasonable request.

## Supplementary Materials

The following supporting information can be downloaded at: https://www.mdpi.com/article/10.3390/cancers17203317/s1, Figure S1: Immunohistochemical (IHC) staining of P53 and CD44 in six representative patient samples. Upper panels show IHC images for P53 and CD44 expression across the six patients. The lower panel summarizes the clinical stage of the lesions along with the quantified percentage of P53- and CD44-positive cells for each patient; Table S1: List of mouse primers and probes used for qPCR analysis. Figure S2: Representative phase-contrast images show oviductal epithelial cells clones that are deficient in *Trp53* and *Brca1* after AdCre treatment exhibit elongated, spindle-shaped morphology consistent with epithelial-to-mesenchymal transition (EMT), indicating enhanced mesenchymal characteristics upon loss of *Trp53* and *Brca1*. Figure S3: Original images of representative agarose gels and Western blots showing the amplified recombinant fragments and protein expression of BRCA1 and P53.
