# Supplementary material for: P53 Mutation Induces Epithelial-to-Mesenchymal Transition (EMT) Associated with Stem Cell Properties and Tumorigenesis in Fallopian Tube Cells
Source: Cancers (Basel). 2025 Oct 14;17(20):3317. doi: 10.3390/cancers17203317 (PMC12564701; doi:10.3390/cancers17203317)

Supplementary Table S1: List of mouse primers and probes used for qPCR analysis.

Gene	Primer or Probe Sequence	Source
Cdh1	[F], 5’-atcctcgccctgctgatt-3<br>[R], 5’-accaccgttctcctccgta-3’	Invitrogen
Krt19	[F], 5’-AATGGCGAGCTGGAGGTGAAGA<br>[R], 5’CTTGGAGTTGTCAATGGTGGCAC	Invitrogen
Pax2	[F], 5’-GGCATCTGCGATAATGACACA-3’<br>[R], 5’-GTGGAAAGGCTGCTGAACTT-3’)	Invitrogen
Pax8	[F], 5’-GCAGCTATGCCTCTGCTA-3’<br>[R], 5’-GCTGTAGGCATTGCCAGAAT-3’	Invitrogen
Ovgp1	[F], 5’-TGCCTCAATGGGACCAGCATCT<br>[R], 5’-TCATAGCCAAGCCACTCCTTCC	Invitrogen
Foxj1	5’-/56-FAM/AGAATTCCA/ZEN/TCCGCCACAACCTGT//3IABkFQ//-3’	IDT
Ly6a	(5’-/56-FAM/ATCTTTGCT/ZEN/ TACCCATCTGCCCTCC/3IABkFQ/-3’)	IDT
Cd44	(5’-/56-FAM/TCTTCTGCC/ZEN/ CACACCTTCTCCTACT/3IABkFQ/-3’)	IDT
Prom1	(5’-/ /56-FAM/CCG ATG CCA /ZEN/TCC AGG TCT GAG AA/3IABkFQ/-3’)	IDT
Aldh1	(5’-/56-FAM/AGTTAACCC/ZEN/ ACACCACCCCAGC/3IABkFQ/-3’)	IDT
Snai1	[F], 5’-GTCTGCACGACCTGTGGAA-3’<br>[R], 5’-CAGGAGAATGGCTTCTCACC-3’	Invitrogen
Trp53	[F], 5’-ATGTGCACGTACTCTCCTCC-3’<br>[R], 5’-ATGGGAGCTAGCAGTTTGGG-3’	Invitrogen
Brca1	[F], 5’-AGGCTTGACCCCCAAAGAAG-3’<br>[R], 5’-GTGTCCGCTCACACACAAAC-3’	Invitrogen
Tbp	[F],5’-CTCAGTTACAGGTGGCAGCA-3’<br>[R]:5’ACCAACAATCACCAACAGCA-3’	Invitrogen
Pipa	[F], 5’- AGGGTGGTGACTTTACACGC-3<br>[R], 5’- GATGCCAGGACCTGTATGCT-3	Invitrogen

A.

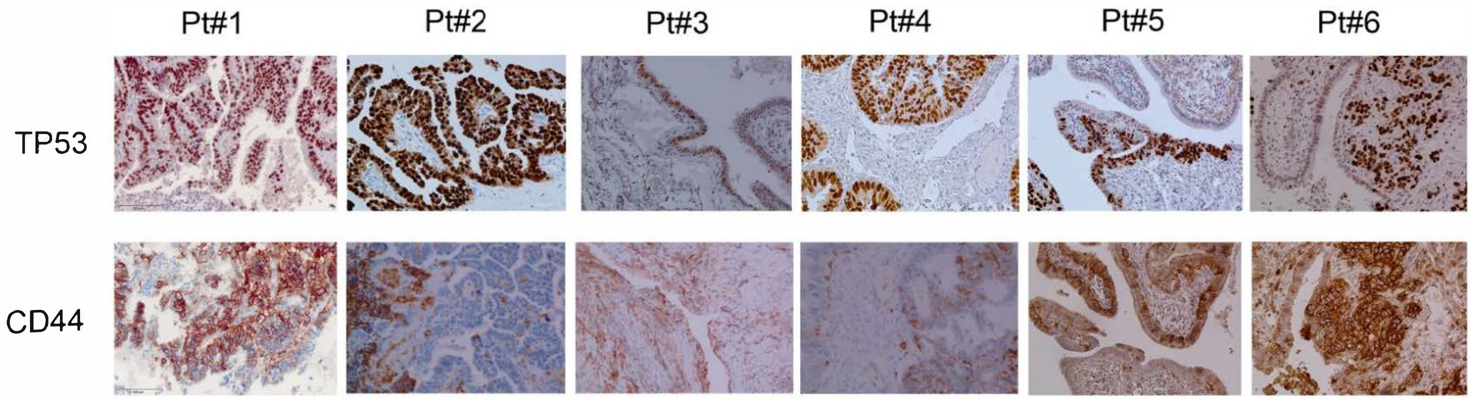

B.

Patients	Type of lesion	% P53	%CD44
Pt#1	Late lesion	37.5	16
Pt#2	Late Lesion	37.211	11.414
Pt#3	Early Lesion	1.101	14.274
Pt#4	Early Lesion	11.774	3.451
Pt#5	Early Lesion	7.171	12.994
Pt#6	Late Lesion	10.931	15.196

Supplementary Figure S1. Immunohistochemical (IHC) staining of TP53 and CD44 in six representative patient samples. Upper panels show IHC images for TP53 and CD44 expression across the six patients. The lower panel summarizes the clinical stage of the lesions along with the quantified percentage of TP53- and CD44-positive cells for each patient.

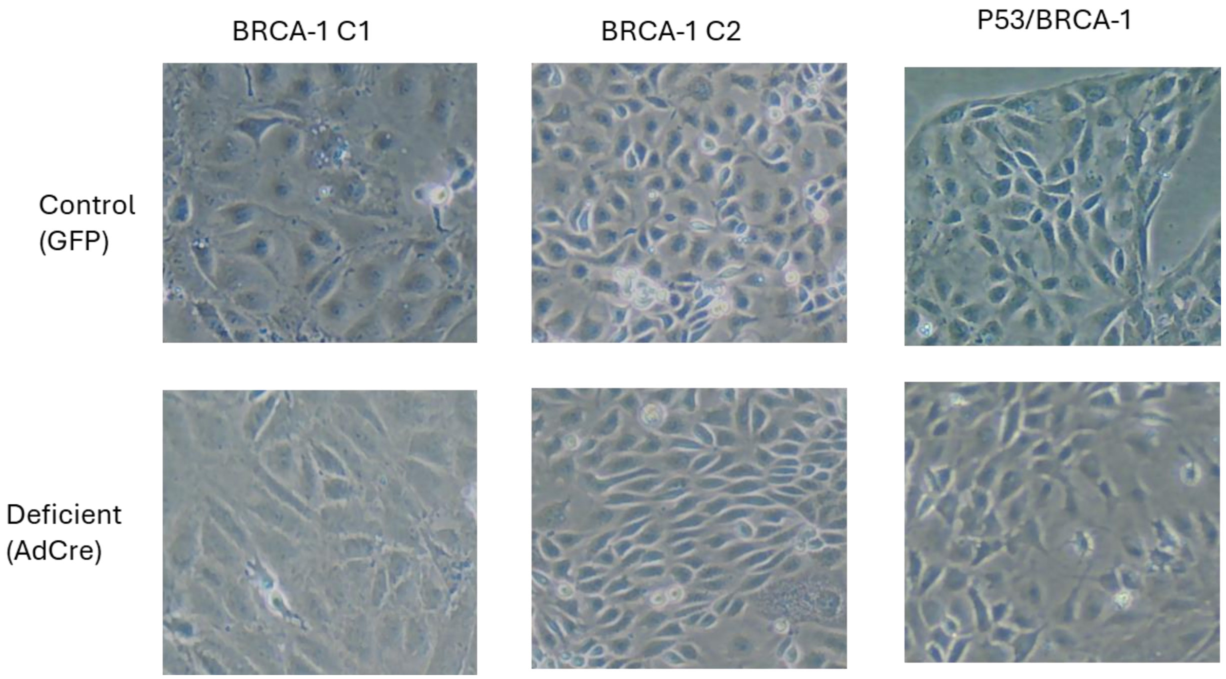

Supplementary Figure S2: Representative phase-contrast images show oviductal epithelial cells clones that are deficient in *Trp53* and *Brca1* after AdCre treatment exhibit elongated, spindle-shaped morphology consistent with epithelial-to-mesenchymal transition (EMT), indicating enhanced mesenchymal characteristics upon loss of *Trp53* and *Brca1*.

Supplementary Figure S3: Original images of representative agarose gels and Western blots showing the amplified recombinant fragments and protein expression of BRCA and P53.

P53 Knockout, PCR and Gel electrophoresis

Figure 2A left panel

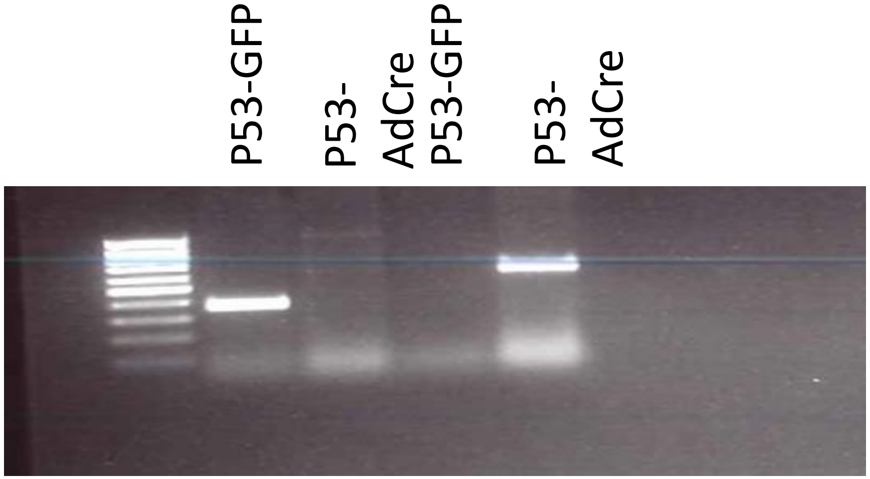

P53
Recombination

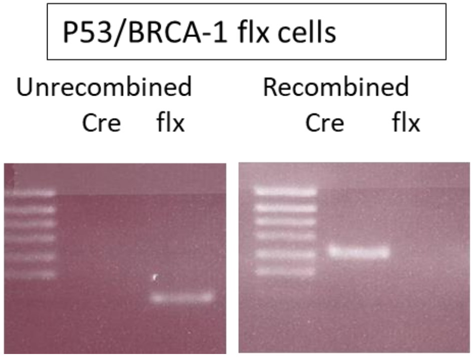

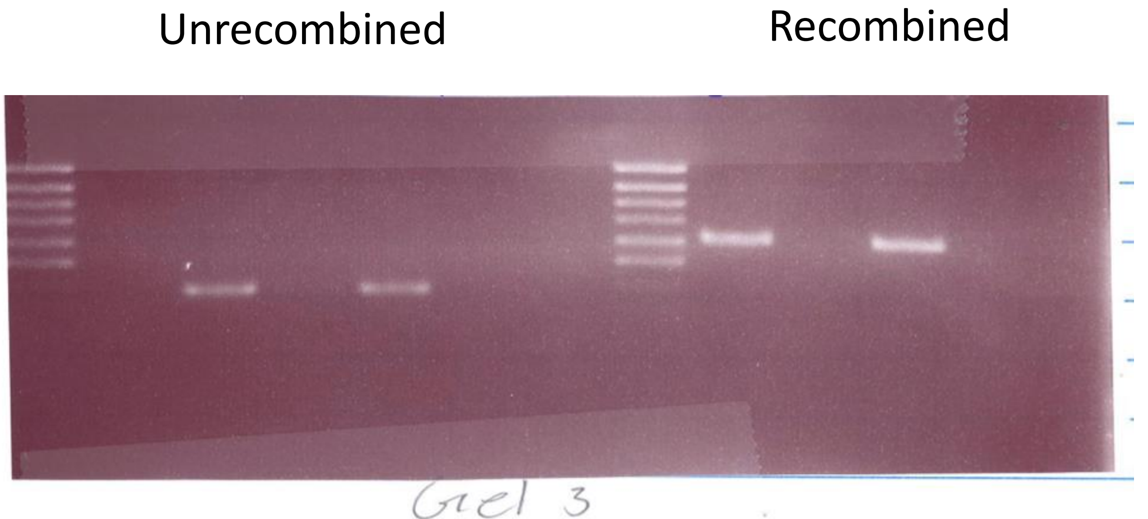

BRCA-1 knockout PCR-Gel electrophoresis

Figure 2A
Right Panel

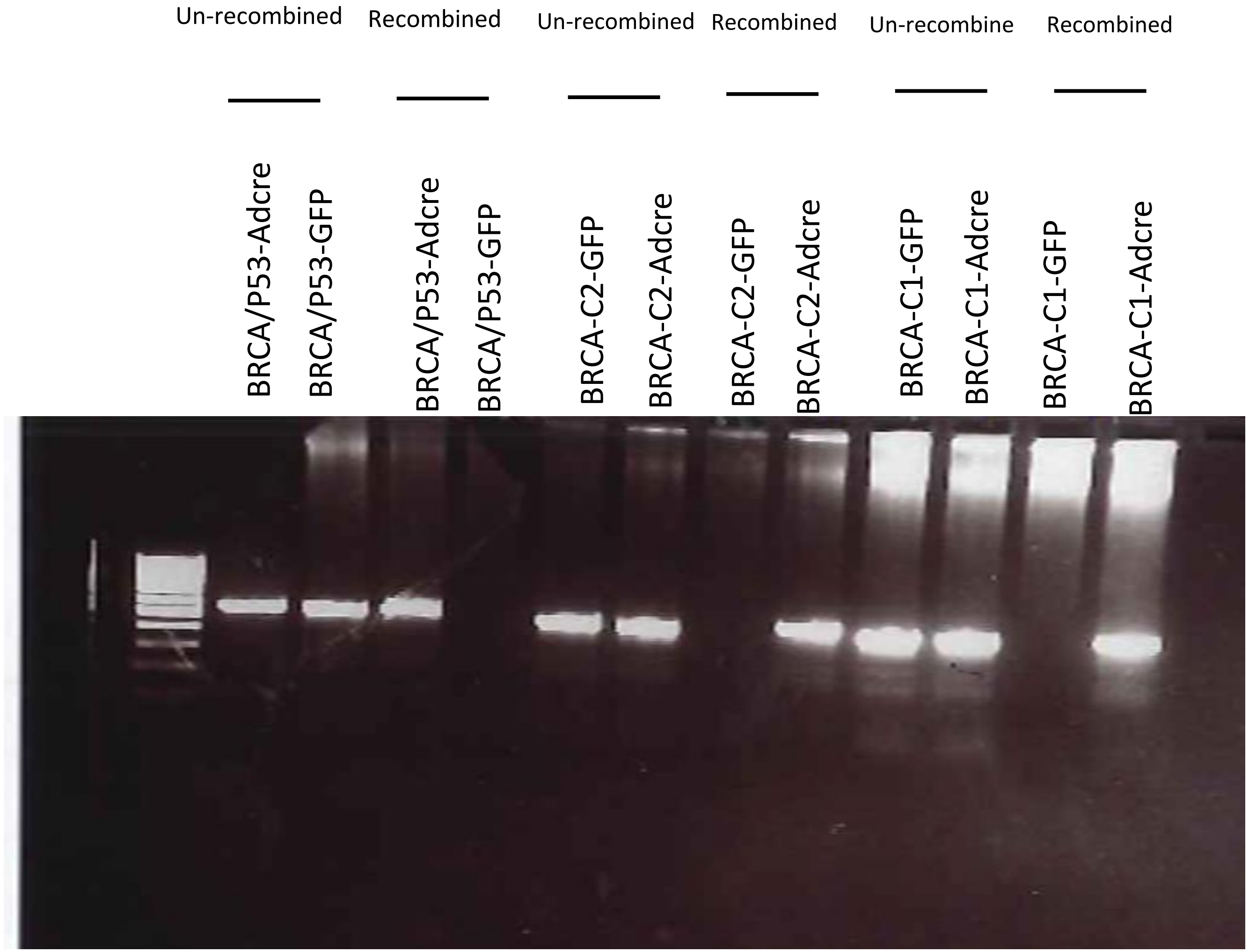

BRCA-1 sequencing

Sample	Primer
1- BRCA-1/flx- C1-GFP	(4F/R)
2-BRCA-1/flx- C1-GFP	(13F/R)
3-BRCA-1/flx- C1-GFP	(4F/R)
4-BRCA-1/flx- C1-GFP	(13F/R)

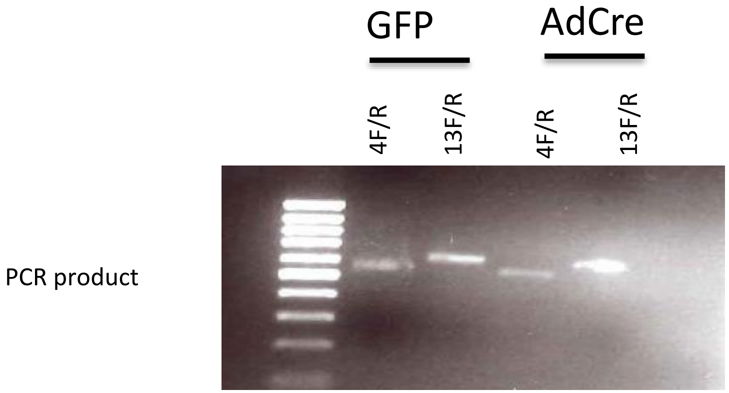

-pcr products are specific for BRCA1

- The loxP sites are in intron 4 and 13

BRCA western blot

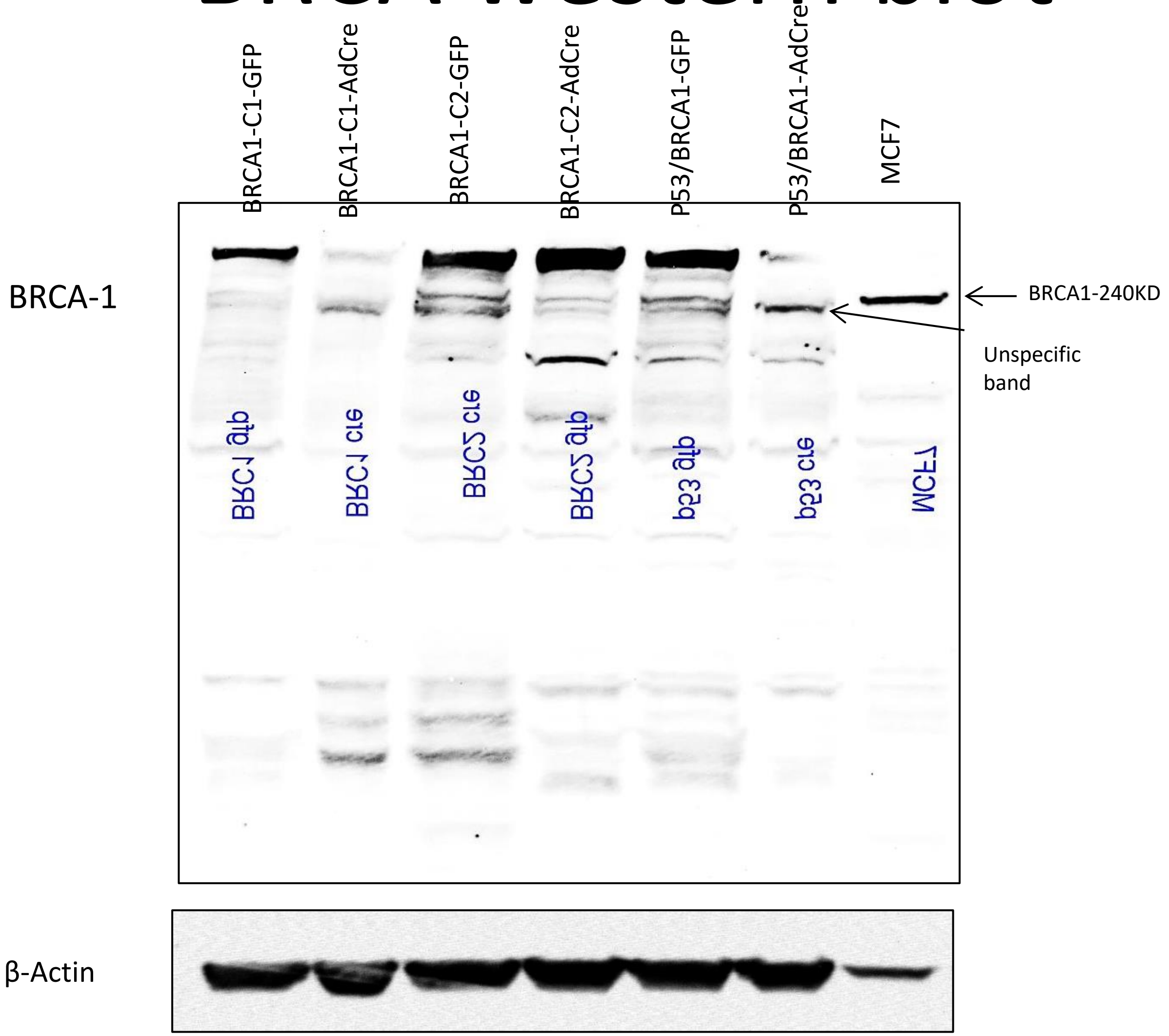

P53 Expression using Western blot

Figure 2B Left Panel

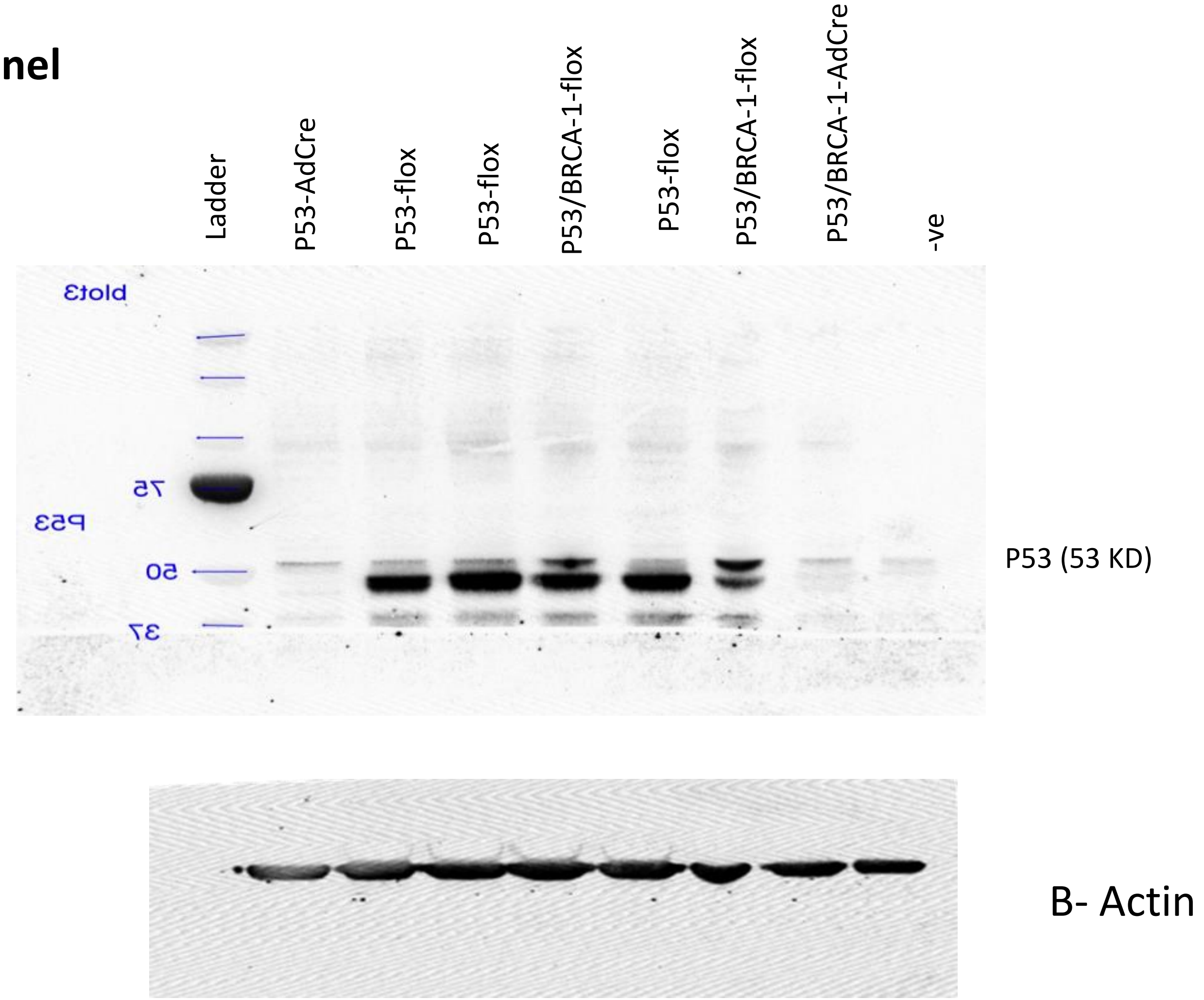

Supplement: Supplementary file 1 [file cancers-17-03317-s001.zip › cancers-3882690-supplementary.pdf]
